# Experimental Characterization of the Interaction between the N-Terminal SH3 Domain of Crkl and C3G

**DOI:** 10.3390/ijms222413174

**Published:** 2021-12-07

**Authors:** Livia Pagano, Francesca Malagrinò, Caterina Nardella, Stefano Gianni, Angelo Toto

**Affiliations:** Istituto Pasteur—Fondazione Cenci Bolognetti, Dipartimento di Scienze Biochimiche “A. Rossi Fanelli” and Istituto di Biologia e Patologia Molecolari del CNR, Sapienza Università di Roma, 00185 Rome, Italy; livia.pagano@uniroma1.it (L.P.); francesca.malagrino@uniroma1.it (F.M.); caterina.nardella@uniroma1.it (C.N.)

**Keywords:** kinetics, site-directed mutagenesis, stopped-flow

## Abstract

Crkl is a protein involved in the onset of several cancer pathologies that exerts its function only through its protein–protein interaction domains, a SH2 domain and two SH3 domains. SH3 domains are small protein interaction modules that mediate the binding and recognition of proline-rich sequences. One of the main physiological interactors of Crkl is C3G (also known as RAPGEF1), an interaction with key implications in regulating cellular growth and differentiation, cell morphogenesis and adhesion processes. Thus, understanding the interaction between Crkl and C3G is fundamental to gaining information about the molecular determinants of the several cancer pathologies in which these proteins are involved. In this paper, through a combination of fast kinetics at different experimental conditions and site-directed mutagenesis, we characterize the binding reaction between the N-SH3 domain of Crkl and a peptide mimicking a specific portion of C3G. Our results show a clear effect of pH on the stability of the complex, due to the protonation of negatively charged residues in the binding pocket of N-SH3. Our results are discussed under the light of previous work on SH3 domains.

## 1. Introduction

Crkl is a ubiquitously expressed 39 kDa adapter protein, member of the proto-oncogene CRK family, that mediates and regulates the linking of several signaling proteins. It was originally discovered in cells from patients with chronic myelogenous leukemia, and its overexpression is correlated with the onset of a number of cancer diseases (recently reviewed in [1]). Crkl plays an essential role in regulating several physiological pathways linked to cytoskeletal changes and cell migration and possesses a prominent role in the onset of human cancers, such as, for example, chronic myelogenous leukemia [2]. Intriguingly, Crkl possesses no catalytic or transcriptional activity and exerts its functions through its protein–protein interaction modules that compose the entire protein, i.e., one N-terminal SH2 domain followed by two SH3 domains (namely N-SH3 and C-SH3).

SH3 domains are small protein interaction modules composed of a five strands β-sandwich and a 3_10_ helix, that typically mediate the binding and recognition of proline-rich sequences, in particular those characterized by the P-X-X-P consensus (X being any amino acid) [3,4], although atypical binding sequences interacting with SH3 have been identified [5,6]. Importantly, the P-X-X-P motif can be arranged into two opposite orientations, defined by the formation of a salt bridge between a positively and a negatively charged residue(s) on the SH3 binding surface [7]. Crk and Crkl display an overlapping list of cellular interactors (such as for example SOS, EPS15 and C3G) [8], which may be explained by their ability to recognize and bind similar consensus sequences P-X-L-P-X-K (Proline-X-Leucine-Proline-X-Lysine) [9,10].

C3G (also known as RAPGEF1, Rap guanine nucleotide exchange factor 1) is the first guanine nucleotide exchange factor discovered to interact with the SH3 domain of CRK [11,12] and to activate Rap1 GTPases [12]. Rap1 regulates several physiological pathways in the cell, ranging from growth and differentiation to cell morphogenesis and adhesion processes [13]. C3G is a small GTPase with a key role in cell adhesion and cell–cell junction formation [14]. C3G is characterized by a specific region that catalyzes the exchange reaction and several polyproline regions conforming to the consensus P-X-X-P-X-K, which allows it to be recognized and bound by SH3 domain containing proteins. Crkl interacts with C3G through its N-terminal SH3 domain. The association of the Crkl-C3G complex with proteins characterized by the presence of a phosphorylated tyrosine has been proposed as basis of the phosphorylation of a specific tyrosine of C3G and consequent activation of Rap1 [15,16]. A lysine residue in position +2 to the P-X-X-P motif has been demonstrated to be of key importance for binding specificity with Crk [17,18]. Structural analysis of the complex reported that the positive charge carried by the lysine is coordinated by three negatively charged residues in the binding pocket of Crk. Those acidic residues are conserved in Crkl.

Because of its involvement in the onset of several cancer pathologies and its functions solely based on mediating protein-protein interactions, a deep understanding of the mechanism of binding of Crkl with its ligands is of primary importance as a first step toward the definition of potential therapeutic strategies aimed to modulate those interactions. In this paper we characterize the binding reaction occurring between the N-terminal SH3 domain of Crkl and a peptide mimicking a specific region of C3G, ranging from residue 277–296, namely C3G_277–296_, (sequence VVDNSPPPALPPKKRQSAPS) through a combination of fast kinetic binding experiments conducted at different experimental conditions and site-directed mutagenesis. Our kinetic analysis demonstrates a clear effect of pH on the stability of the complex, allowing us to ascribe this effect on the protonation of negatively charged residues. By removing these negative charges through site-directed mutagenesis, we could characterize their specific role in the binding event. Our results and their implications are then discussed under the light of previous work on SH3 domains.

## 2. Results and Discussion

### 2.1. The Recognition Event of C3G by the N-SH3 Domain Is Electrostatically Driven

In order to characterize the binding reaction between the N-SH3 domain of Crkl and C3G, we conducted kinetic binding experiments with a stopped-flow apparatus, by rapidly mixing the N-SH3 domain versus the C3G_277–296_ peptide, the latter covalently linked with a dansyl group at its N-terminus (Dansyl-VVDNSPPPALPPKKRQSAPS). This modification allowed us to monitor the binding reaction by following the change of Förster resonance energy transfer (FRET) signal upon binding, which generates from the two naturally present tryptophan residues in the N-SH3 domain in position 164 and 165 (donor) and the dansyl group linked to the peptide (acceptor).

To characterize the binding between N-SH3 and the C3G_277–296_ peptide, we resorted to carry out pre-steady state rapid mixing stopped-flow experiments. In these types of experiments, a common practice lies in performing the kinetic analysis under the so-called pseudo-first-order conditions, i.e., a condition in which one of the two reactants is held at a much higher concentration than the other. In practice, in many cases, it is extremely difficult to achieve such conditions [19]. This is particularly true in cases in which the observed rate constants are so high to approach the experimental limitations of the stopped-flow apparatus. Accordingly, as described below, the analysis of the binding data must be performed by applying the analytical solution of the bimolecular binding transition [19,20].

**Figure 1:**
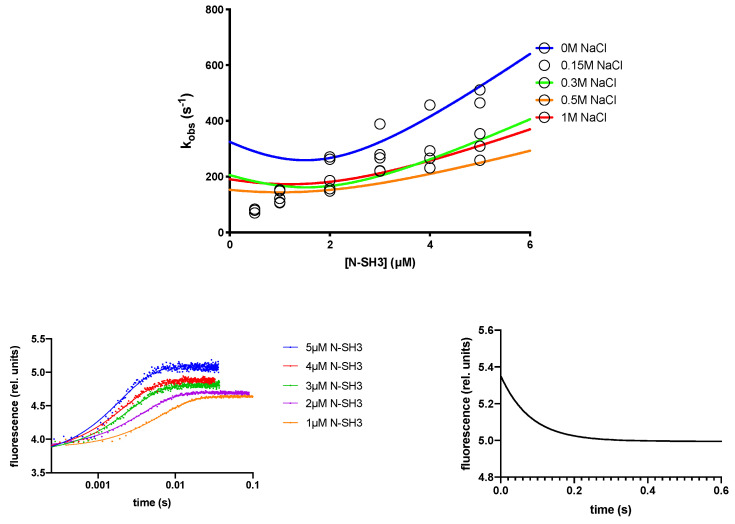
(**Top**) Kinetic binding experiments performed by mixing a constant concentration of dansylated C3G_277–296_ peptide (0.5 µM) versus increasing concentrations of N-SH3 in buffers containing different NaCl concentrations (see legend). Lines are the best fit to Equation (1). (**Bottom left**) Average kinetic traces obtained in binding experiments between dansylated C3G_277−296_ peptide and different concentration of N-SH3 at different concentrations in buffer Hepes 50 mM pH 7.0. Lines are the best fit to a single-exponential equation. (**Bottom right**) Average displacement kinetic trace obtained mixing a preincubated complex of N-SH3 domain and dansylated C3G_277−296_ peptide versus a high excess of nondansylated C3G_277−296_ peptide in buffer Hepes 50 mM pH 7.0. Line represents the best fit to a single-exponential equation.

We rapidly mixed a constant concentration of C3G_277–296_ peptide (0.5 µM) versus increasing concentrations of N-SH3 (ranging from 0.5 to 5 µM) and the observed rate constants (*k*_obs_) obtained at different ionic strength conditions (buffer Hepes 50 mM, pH 7.0, 0.15 M, 0.3 M, 0.5 M and 1 M NaCl) were plotted as function of concentration of N-SH3 and fitted with a linear equation (Figure 1). By following previously derived equations [19,20], the dependence of *k*_obs_ as function of [N-SH3] was fitted with Equation (1), taking into account the non-pseudo-first order conditions [19], allowing us to calculate the microscopic association rate constant (*k*_on_), the microscopic dissociation rate constant (*k*_off_) of the binding reaction and the equilibrium dissociation rate constant *K*_D_, such as *k*_off_/*k*_on_. To increase the reliability of the calculated *K_D_*, we resorted to directly obtaining a *k*_off_ value through displacement experiments (Table 1), in which a preincubated complex of N-SH3 domain and dansylated C3G_277–296_ peptide (at the concentration of 0.5 and 2 µM, respectively) were rapidly mixed versus a high excess of nondansylated peptide (ranging from 20 to 40 µM). In agreement with the theory in [21], the observed rate constants were insensitive to displacer concentration. In all binding and displacement experiments conducted, traces were satisfactorily fitted with a single-exponential equation.

(1) $$k_{obs}={\left(k_{on}{}^{2}*{\left(2-\left[NSH3\right]\right)}^{2}+k_{off}{}^{2}+2k_{on}k_{off}\left(2+\left[NSH3\right]\right)\right)}^{1/2}$$

It should be noticed that the observed rate constants reported in Figure 1 are at the limit of the experimental detection by stopped-flow experiments. Consequently, all the binding experiments reported in this work were measured at 10 °C, in order to slow down the apparent transitions. Hence, it is important to note that the experimental conditions significantly deviate from the physiological conditions, and their interpretation should be mainly taken with comparative purposes.

Changing the ionic strength of the solution is the simplest way to modulate an electrostatically driven binding reaction. It is generally known that shielding the electrostatic attraction of diffusion controlled binding reactions leads to the opposite effects on the microscopic association and dissociation rate constants, with *k*_on_ rapidly decreasing upon increasing ionic strength [22]. In in vitro experiments, the interaction between two proteins is the result of a random collision forming an encounter complex, stabilized in a final complex after one (or more) transition state(s). In such scenarios, the early events of recognition between charged residues of the two proteins are diffusion controlled and affected by ions dissolved in solution. Then, the binding follows, with desolvation of polar and charged residues at the interface between the two proteins, the bound complex remaining mostly unaffected by increasing concentration of salt in solution. Accordingly, in the case of N-SH3 domain binding with C3G_277–296_, the inspection of Figure 1 and the analysis of kinetic data at different ionic strength conditions (reported in Table 1) display a pronounced effect of salt concentration on the *k*_on_.

### 2.2. Protonation of Negatively Charged Residues Abolishes Binding

**Figure 2:**
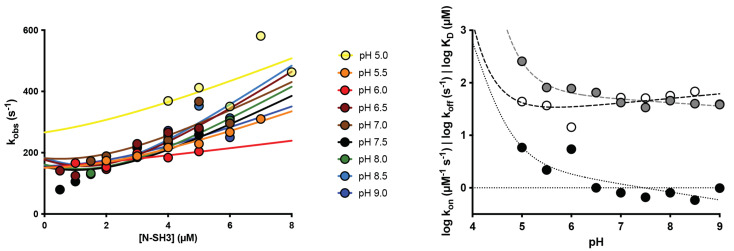
(**Left**) Kinetic binding experiments performed by mixing a constant concentration of dansylated C3G_277–296_ peptide (0.5 µM) versus increasing concentrations of N-SH3 at different pH conditions (see legend). Lines are the best fit to Equation (1). (**Right**) Dependence of logarithm of *k*_on_ (white) and *k*_off_ (gray) and equilibrium dissociation rate constant (black) as a function of pH. Lines are the best fit to Henderson–Hasselbalch equation.

To further investigate the role of charges in the binding reaction between N-SH3 domain and C3G, we resorted to performing kinetic binding experiment at different pH conditions, at a range of pH between 5.0 and 9.0. The observed rate constants obtained at different pH conditions were fitted with Equation (1) (Figure 2, left), and the calculated kinetic parameters *k*_on_ and *k*_off_ (obtained from separated displacement experiments) are reported in Table 1.

The dependence of *k*_on_ and *k*_off_ as function of pH, reported in Figure 2, right, clearly shows that the affinity between the two interacting molecules decreases with decreasing pH. Unfortunately, the very high value of *k*_off_ prevents any reliable analysis using a Henderson–Hasselbalch equation. In fact, in the case of the N-SH3, we could not obtain a sigmoidal profile, due to the impossibility to measure binding at pH < 5.0. Although the beginning of a transition is clearly visible, an accurate fit would require more data points for pH < 5.0. On the basis of the available data, it may be concluded that acidic pH conditions cause a dramatic increase in the *k*_off_, with the *k*_on_ being less affected, determining a pronounced destabilization of the complex.

**Figure 3:**
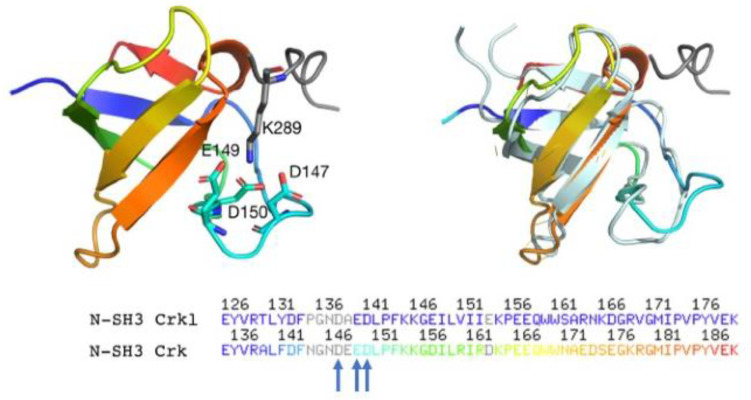
**Left**: three-dimensional structure of the N-SH3 domain of Crk (rainbow colors) in complex with C3G (gray) (PDB: 1cka). Acidic residues D147, E149 and D150 together with basic residue K289 of C3G are highlighted in sticks. K289 is coordinated by the three negatively charged residues in the binding pocket of Crk, which are conserved in Crkl; **right and bottom**: structural and sequence alignment of the N-SH3 domain of Crk (rainbow colors) with the N-SH3 domain of Crkl (light blue). D147, E149 and D150 residues of Crk are highlighted with blue arrows.

The analysis of these data provides us with important information about the binding mechanism of the N-SH3 domain with C3G. In light of what was previously shown for the ionic strength dependence, kinetic data obtained at different pH highlight a double role for salt bridges formation in both the early recognition events and the stabilization of the complex. Moreover, since SH3 domains have evolved to recognize and bind polyproline sequences, and proline binding occurs mainly through C-H·π interactions with aromatic residues [23,24], the formation of canonical salt bridges may give a substantial contribution in optimizing the recognition and binding of the substrate, improving the specificity in the crowded intracellular environment. Interestingly, the comparison of the primary structures of Crk and Crkl highlights the conservation of three negatively charged residues (D147, E149 and D150 on Crk, D138, E140 and D141 on Crkl) physically located at the binding interface of the proteins (Figure 3). Based on this evidence and on previous structural work on Crk [18], all together our results suggest that D138, E140 and D141 residues of the N-SH3 domain of Crkl may be responsible for a salt bridge formation with a positively charged residue on C3G.

### 2.3. Determining the Role of D138, E140 and D141 by Site-Directed Mutagenesis

**Figure 4:**
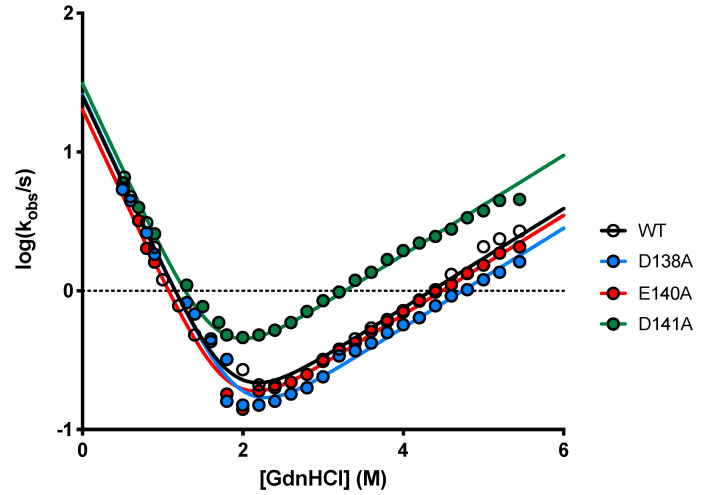
Dependence of the logarithm of the observed rate constants for unfolding and refolding experiments as a function of [GdnHCl] for WT, D138A, E140A and D141A (see legend) obtained in buffer Hepes 50 mM pH 7.5 at 25 °C. Data were globally fitted by sharing kinetic m-values. Lines are the best fit to an equation describing a two-state folding mechanism. WT—*k*_f_ = 25.3 ± 2.0 s^−1^, *k*_u_ = 0.027 ± 0.002 s^−1^; D138A—*k*_f_ = 25.9 ± 2.2 s^−1^, *k*_u_ = 0.019 ± 0.001 s^−1^; E140A—*k*_f_ = 20.2 ± 1.6 s^−1^, *k*_u_ = 0.024 ± 0.001 s^−1^; D141A—*k*_f_ = 31.2 ± 2.7 s^−1^, *k*_u_ = 0.066 ± 0.004 s^−1^; globally shared m-values—m_f_ = 1.68 ± 0.04 kcal mol^−1^ M^−1^, m_u_ = 0.49 ± 0.01 kcal mol^−1^ M^−1^.

In an effort to analyze the mechanistic role of the residues D138, E140 and D141 in the binding reaction with C3G, we performed site-directed mutagenesis and generated the D138A, E140A and D141A variants of the N-SH3 domain. At first, to monitor the effect of these mutations on the stability of the domain, we performed (un)folding kinetic experiments in buffer Hepes 50 mM pH 7.0 at 25 °C. The dependence of the logarithm of the observed rate constants (*k*_obs_) obtained at different [GdnHCl] (chevron plot) for the wild-type, and the three variants are reported in Figure 4. All the chevron plots were globally fitted by sharing kinetic m-values [25] with Equation (2) describing a two-state scenario, suggesting the absence of intermediate(s) populating along the reaction pathway [26,27]. Importantly, none of the three mutations cause a disruption of the native state, albeit D141A mutation appears mildly destabilizing compared to D138A and E140A.

(2) $$k_{obs}=k_{f}^{0}exp\left(-m_{f}\left[GndHCl\right]/RT\right)+k_{u}^{0}exp\left(m_{u}\left[GndHCl\right]/RT\right)$$

**Figure 5:**
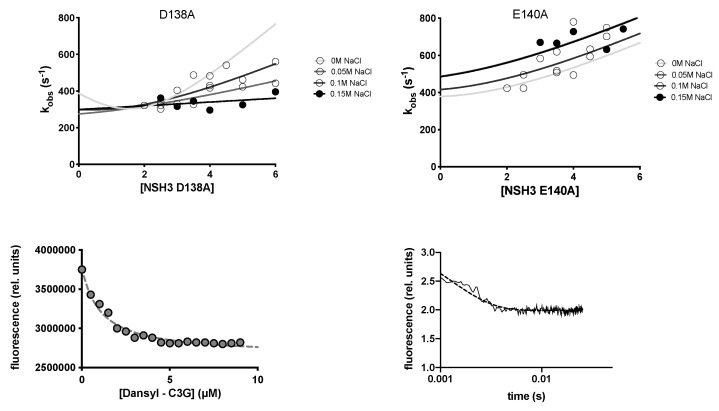
(**Top left**) Kinetic binding experiments performed by mixing a constant concentration of dansylated C3G_277–296_ peptide (0.5 µM) versus increasing concentrations of N-SH3 D138A at different ionic strength conditions (see legend). Lines are the best fit to Equation (1). (**Top right**) Kinetic binding experiments performed by mixing a constant concentration of dansylated C3G_277–296_ peptide (0.5 µM) versus increasing concentrations of N-SH3 E140A at different ionic strength conditions (see legend). Lines are the best fit to Equation (1). (**Bottom left**) Equilibrium binding experiment between N-SH3 D141A held at constant concentration and increasing concentrations of dansylated C3G_277–296_ peptide. Line is the best fit to a hyperbolic function. (**Bottom right**) Average displacement trace obtained for D141A. See text for details of conditions used. Lines are the best fit to a single-exponential function.

Then, we employed D138A, E140A and D141A variants in kinetic binding experiments with C3G, and we explored the effect of increasing ionic strength on the binding reaction of these variants. The results obtained are reported in Figure 5, and the calculated kinetic data are listed in Table 2. The inspection of Figure 5 and analysis of kinetic data highlight the D138A variant to be affected by increasing salt concentrations, while E140A shows no evident effects. A comparison of kinetic data obtained in the absence of NaCl shows that whilst the microscopic association rate constants calculated for D138A and E140A variants is comparable with the one obtained for the wt, an increase in *k*_off_ is appreciable. All these aspects suggest a key role of E140 in the recognition event, with D138A being involved mainly in the late events. This scenario is further confirmed by the evidence of a rapidly decreasing *k*_on_ upon increasing ionic strength of the solution for D138A variant, with the electrostatic charges carried by D138 residue not being involved in the recognition of a positively charged residue on C3G. On the other hand, the overall absence of effect of salt dependence on binding kinetics upon removal of E140 negative charge suggests the interactions formed by this residue acting as a prominent determinant of the early events of the binding reaction between the N-SH3 domain and C3G.

It is of particular interest to discuss the effect of D141A mutation. When employed in kinetic binding experiments, this variant did not return any measurable trace describing a change in FRET signal upon mixing, possibly due to an overall destabilization of the complex and/or sub-millisecond kinetics that could not be resolved by the stopped-flow. To further investigate this aspect and obtain a quantitative measure of the binding affinity of D141A variant with C3G, we resorted to conduct equilibrium binding experiments. Experiments were conducted by exciting samples containing a constant concentration (1 µM) of N-SH3 D141A at 280 nm and following the progressive quenching of two tryptophan residues (in position 164 and 165) fluorescence emission at increasing concentrations of dansyl-C3G_277–296_. The dependence of fluorescence measured at 350 nm as function of dansyl-C3G concentration is reported in Figure 5. Interestingly, the fitting of data with a hyperbolic equation returned a *K*_D_ = 1.07 ± 0.05 µM, demonstrating that D141A variant is capable of binding. On the other hand, although this value reflects a relatively high affinity in the low µM range, it appears to be 10-fold higher than what was measured about wt from kinetic data (*k*_off_/*k*_on_= *K*_D_ = 0.09 ± 0.05 µM). Such a decrease in binding affinity may be at the basis of our impossibility to time-resolve the binding reaction with a stopped-flow apparatus, with the reaction possibly occurring in the dead time of the instrument.

To further investigate this aspect, we resorted to performing displacement experiments, targeted to the direct calculation of microscopic dissociation rate constant *k*_off_. We challenged a preincubated complex of D141A NSH3 domain (2 µM) and dansyl-C3G_277–296_ (10 µM) versus a high excess of nondansylated C3G (50 µM), and we could not measure any change in fluorescence emission, suggesting the *k*_off_ being too high to be measured at the stopped-flow. To test this and to slow down the diffusion of molecules, we repeated the experiment increasing the viscosity of the solution adding 20% *w*/*v* sucrose to the buffer Hepes 50 mM pH 7.0. As expected, the increase in solution viscosity allowed us to obtain a displacement trace (shown in Figure 5 bottom right panel). Although the trace was satisfactorily fitted with a single-exponential equation, the *k*_off_ calculated is 800 ± 30 s^−1^, a value that is far beyond the resolution capability of the stopped-flow, a consistent part of the reaction occurring in the dead time of the instrument. Based on the calculated affinity of D141A variant for C3G in the absence of sucrose, this dramatic increase in *k*_off_ might be accompanied by a strong increase in *k*_on_.

It is of particular interest to compare the effect of the three site-directed mutants described in this work. In fact, whilst a specific role in the early and late events of the binding reaction could be determined for D138 and E140 residues, in the case of D141, both microscopic association and dissociation rate constants were affected upon mutation. Hence, whereas D138 plays a key role in the stabilization of the formed complex and E140 is mainly involved in the early recognition events of the binding reaction, the D141 sidechain appears to play a key role in both events, in particular in the formation of electrostatic interactions occurring downhill the main energy barrier of the reaction. The increase in *k*_on_ which must occur in order to maintain a relatively high affinity suggests that the absence of the negative charge in position 141 strongly improves the early recognition event of C3G. Overall, our data support a scenario in which, aside from the nonspecific ionic interactions occurring between N-SH3 and C3G, an additional specific step occurs, driven at least in part by D141 residue, contributing to the final stabilization of the formed complex.

### 2.4. Determinants of N-SH3 Domain Binding Selectivity

SH3 domains are widespread protein–protein interaction domains. Their main biochemical property relies in the recognition of proline rich sequences, generally identified with the P-X-X-P consensus. Given their fundamental importance in many physiological and molecular pathways in the eucaryotic cell and their role in several human pathologies, SH3 domain gained a strong attention of the scientific community since their discovery, and many of them have been characterized in their mechanisms of interaction with their ligands [4,28,29,30,31,32]. The general structural properties of the recognition of ligands by SH3 domains are well established [30]. However, understanding the molecular determinants of specificity of SH3 domains in general is a difficult task to address, given the large amount of atypical consensus sequences that have been discovered [28].

Our group previously described in detail the mechanism of interaction of the C-SH3 domain of Grb2 with Gab2 [31,32], which is regulated by a complex allosteric mechanism. An analysis of the structure of C-SH3:Gab2 complex (PDB: 2vwf) highlights the presence of negatively charged residues in the binding pocket in direct contact with basic residues of Gab2. Importantly, the topological distribution of these negative charges appears conserved between the C-SH3 domain of Grb2 and the N-SH3 domain of Crk. Ionic strength and pH dependence analysis of the binding reaction, together with mutational analysis presented in this paper, highlight a prominent role of D138, E140 and D141 residue of Crkl in the binding of C3G_277–296_. Our data show E140 being mainly involved in the early events of recognition of a positively charged residue carried by C3G and D138 with a stabilizing effect on the formed complex. Although we could not resolve kinetics for D141A variant, the data obtained from equilibrium binding experiments and displacement experiments show an effect on binding affinity. In analogy to what was previously found for the N-SH3 domain of Crk [18] and in light of the structural and sequence alignment (Figure 3) our data suggest that D138, E140 and D141 residues may coordinate K289 residue of C3G through salt bridges formation. This electrostatic attraction driving this fundamental protein–protein interaction may represent a key aspect of a conserved mechanism of binding in the Crk family, although on the other hand, it raises questions about how promiscuity is avoided in the intracellular milieu. Future work based on structural determination of the N-SH3:C3G complex and on extensive site-directed mutagenesis would allow us to characterize the selectivity determinants of the N-SH3 domain of Crkl, determine which specific positive residue on C3G plays a role in the recognition, and pinpoint possible long-range allosteric regulation of the binding (described also for other small protein–protein interaction modules, such as PDZ domains [33,34]) occurring simultaneously and/or finely tuning the binding interface of the domain.

## 3. Conclusions

Achieving a deep understanding of the interaction occurring between Crkl protein and its ligands is of fundamental importance to gaining useful information about the molecular basis of several physiological pathways and human pathologies in which this protein is involved. The employment of rigorous kinetic characterization of the binding reaction at different experimental conditions, together with site-directed mutagenesis, allowed us to describe in detail the roles of electrostatic forces occurring between the N-SH3 domain and a peptide mimicking one of its physiological partners, C3G. Importantly, whilst the SH3 domains are generally thought to recognize their ligands via the P-X-X-P recognition motif, our data exemplify the existence of a negatively charged stretch in the SH3, which is critical in determining the affinity between the interacting molecules. In this view, our study complements and enriches the structural knowledge on this important protein system, by providing a mechanistic insight on the role of these residues, as probed by the effect of site-directed mutagenesis on the association and dissociation rate constant, respectively. Our data represent a first step for future structural and extensive mutational characterization of this protein system.

## 4. Materials and Methods

### 4.1. Site-Directed Mutagenesis

The construct encoding the N-SH3 domain of Crkl was subcloned in a pET28b+ plasmid vector. The constructs encoding D138A, E140A and D141A were obtained through site-directed mutagenesis using the QuikChange Lightning Site-Directed Mutagenesis kit (Agilent technologies) according to the manufacturer’s instructions. All the mutations were confirmed by DNA sequencing.

### 4.2. Protein Expression and Purification

The expression of all the His-tagged constructs was performed in *E. coli* cells, strain BL21. Bacterial cells were grown in LB medium, with 30 μg/mL of kanamycin, at 37 °C until OD_600_ = 0.7−0.8 and then induced with 0.5 mM IPTG. The cultures were grown at 37 °C for three hours after induction, kept at 25 °C overnight and then collected by centrifugation. Purification was performed resuspending the pellet in 50 mM TrisHCl, 0.3 M NaCl, pH 7.5 buffer with the addition of antiprotease tablet (Complete EDTA-free, Roche), and then sonicated and centrifuged. The soluble fraction from bacterial lysate was loaded onto a nickel-charged His-Trap chelating HP (GE Healthcare) column equilibrated with 50 mM TrisHCl, 0.3 M NaCl and pH 7.5. Protein was then eluted with a gradient from 0 to 0.5 M imidazole by using an ÄKTA-prime system. Fractions containing the protein were collected, and the imidazole was removed using a HiTrap Desalting column (GE Healthcare), with the protein purified in the final buffer of TrisHCl 50 mM, NaCl 0.3 M, pH 7.5. The purity of the proteins was analyzed through SDS-page.

Peptides mimicking the portion of C3G ranging from residue 277 to 296 (sequence VVDNSPPPALPPKKRQSAPS) in their dansylated and nondansylated variants were purchased from GenScript Biotech.

### 4.3. Stopped-Flow (un)folding Experiments

Kinetic (un)folding experiments were performed on an Applied Photophysics Pi-star 180 stopped-flow apparatus, monitoring the change of fluorescence emission, exciting the sample at 280 nm and recording the fluorescence emission by using a 320 nm cutoff glass filter. In all experiments, performed at 25 °C in buffer 50 mM Hepes pH 7.5, refolding and unfolding were initiated by an 11-fold dilution of the denatured or the native protein with the appropriate buffer (0 M and 6 M Guanidine HCl). For each denaturant concentration, at least five individual traces were averaged, and the final protein concentration was 1.5 μM. The fluorescence time courses obtained was satisfactorily fitted by using a single-exponential equation. The chevron plots obtained were fitted using an equation describing a two-state folding mechanism.

### 4.4. Stopped-Flow Kinetic Binding and Displacement Experiments

Kinetic binding experiments were performed on a single-mixing SX-18 stopped-flow instrument (Applied Photophysics), by mixing a constant concentration (0.5 μM) of C3G dansylated versus increasing concentrations of N-SH3 at 10 °C. Ionic strength dependence of wt N-SH3 was performed with concentrations ranging from 1 to 5 µM for 0 M NaCl condition and from 0.5 to 5 µM for 0.15 M, 0.3 M, 0.5 M and 1 M NaCl, buffer Hepes 50 mM pH 7.0. The excitation wavelength was 280 nm, and fluorescence was collected using a 455 nm cut-off filter. At least, five independent acquisitions were collected and averaged for each experiment. The resulting averages were all satisfactorily fitted with a single-exponential equation. Buffers used for pH dependence were: 50 mM Sodium Acetate pH 5.0, 50 mM Sodium Acetate, pH 5.5, Bis–Tris 50 mM pH 6.0, Bis–Tris 50 mM pH 6.5, Hepes 50 mM pH 7.0, Hepes 50 mM pH 7.5, Tris–HCl 50 mM pH 8.0, Tris–HCl 50 mM pH 8.5, Tris–HCl 50 mM pH 9.0, all with added 0.5 M NaCl. Concentrations of N-SH3 used for each experiment were: 4.0–8.0 µM at pH 5.0, 2.0–7.0 µM at pH 5.5, 1.0–5.0 µM at pH 6.0, 0.5–5.0 µM at pH 6.5, 1.5–6.0 µM at pH 7.0, 0.5–5.0 µM at pH 7.5, 1.5–6.0 µM at pH 8.0, 1.5–6.0 µM pH 8.5, 1.5–6.0 µM at pH 9.0. For the ionic strength dependence of D138A and E140A variants buffer used were 0.05 M NaCl, 0.1 M NaCl, 0.15 M NaCl, in 50 mM Hepes pH 7.5. Concentrations of N-SH3 D138A used for each experiment were: 2.0–4.5 µM at 0 M NaCl, 2.5–6.0 µM at 0.05 M, 0.1 M, 0.15 M NaCl. Concentrations of N-SH3 E140A used for each experiment were: 2.0–4.5 µM at 0 M NaCl, 3.0–5.5 µM at 0.05 M NaCl, 3.0–5.0 µM at 0.1 M NaCl, 3.0–5.5 µM at 0.15 M NaCl. Displacement kinetic experiments were performed by mixing a preincubated complex of N-SH3 (in all its variants) and dansylated C3G versus a high excess of nondansylated C3G (see text for details). Experiments were performed in the same buffer conditions as the binding experiments, except for D141A variant, for which 20% *w*/*v* sucrose was added to the buffer Hepes 50 mM pH 7.0. Displacement traces were fitted with a single-exponential equation.

### 4.5. Equilibrium Binding Experiment of D141A

Equilibrium experiment on D141A (fixed concentration at 1 μM) was carried out on a Fluoromax single-photon counting spectrofluorometer (Jobin-Yvon, Newark, NJ, USA), by mixing the construct with increasing dansyl-C3G concentrations. Experiments were performed at 10 °C, using a quartz cuvette with a path length of 1 cm, in 50 mM Hepes pH 7.0 and measuring the change in fluorescence of the naturally present tryptophan residues in position 164 and 165 at increasing concentration of dansyl-C3G. The excitation wavelength was 280 nm, and fluorescence spectra were recorded between 300 and 400 nm.

## Figures and Tables

**Table 1:** Kinetic parameters at different ionic strength conditions and different pH (in presence of 0.5 M NaCl) obtained from linear fitting of data reported in Figure 1 and Figure 2. The NaCl concentrations reported in the left column were added to buffer Hepes 50 mM pH 7.0.

**N-SH3 WT versus C3G_277–296_**			
**[NaCl]**	***k*_on_ (µM^−1^ s^−1^)**	***k*_off_ (s^−1^)**	***K*_D_ (µM)**
**0 M**	130 ± 7	12 ± 1	0.09 ± 0.05
**0.15 M**	96 ± 8	23 ± 1	0.24 ± 0.06
**0.3 M**	83 ± 7	32 ± 2	0.39 ± 0.14
**0.5 M**	51 ± 4	34 ± 1	0.66 ± 0.12
**1.0 M**	68 ± 5	51 ± 2	0.75 ± 0.20

**pH**	***k*_on_ (µM^−1^ s^−1^)**	***k*_off_ (s^−1^)**	***K*_D_ (µM)**
**5.0**	44 ± 15	257 ± 10	5.9 ± 0.8
**5.5**	37 ± 3	81 ± 3	2.2 ± 1.0
**6.0**	15 ± 4	78 ± 3	5.4 ± 1.5
**6.5**	65 ± 4	65 ± 2	1.0 ± 0.6
**7.0**	52 ± 5	42 ± 2	0.8 ± 0.5
**7.5**	51 ± 5	34 ± 2	0.7 ± 0.3
**8.0**	56 ± 5	46 ± 3	0.8 ± 0.4
**8.5**	68 ± 7	40 ± 3	0.6 ± 0.4
**9.0**	39 ± 4	39 ± 2	1.0 ± 0.4

**Table 2:** Kinetic parameters obtained from linear fitting of data reported in Figure 5 (Top left, Top right ). Equilibrium dissociation rate constant *K*_D_ were calculated as *k*_off_/*k*_on_.

**N-SH3 D138A versus C3G_277–296_**			
**[NaCl]**	***k*_on_ (µM^−1^ s^−1^)**	***k*_off_ (s^−1^)**	***K*_D_ (µM)**
**0 M**	158 ± 12	139 ± 1	0.9 ± 0.3
**0.05 M**	81 ± 8	212 ± 2	2.6 ± 0.6
**0.10 M**	48 ± 8	183 ± 2	3.8 ± 0.3
**0.15 M**	12 ± 5	254 ± 2	22.0 ± 2.0
**N-SH3 E140A versus C3G_277–296_**			
**[NaCl]**	***k*_on_ (µM^−1^ s^−1^)**	***k*_off_ (s^−1^)**	***K*_D_ (µM)**
**0 M**	78 ± 6	254 ± 3	3.3 ± 0.7
**0.05 M**	89 ± 8	330 ± 3	3.7 ± 0.6
**0.10 M**	77 ± 8	302 ± 2	3.9 ± 0.8
**0.15 M**	81 ± 8	339 ± 2	4.2 ± 1.0
